# Clinical Features and Familial Mutations in an Autosomal-Inherited Alport Syndrome Patient With the Presentation of Nephrotic Syndrome

**DOI:** 10.3389/fped.2021.678633

**Published:** 2021-11-11

**Authors:** Dahai Wang, Chunrong Shan, Xinxin Jing, Qiuye Zhang, Hong Chang, Yi Lin

**Affiliations:** ^1^Department of Pediatric Cardiology, Nephrology and Rheumatology, The Affiliated Hospital of Qingdao University, Qingdao, China; ^2^Department of Neurology, Qingdao Women and Children's Hospital, Qingdao, China

**Keywords:** Alport syndrome, autosomal inheritance, COL4A4, nephrotic syndrome, end-stage renal disease

## Abstract

**Background:** The aim of this study was to report the clinical features and mutations in a patient with autosomal-inherited Alport syndrome (AS).

**Methods:** We examined the clinical data, mutation analysis results, and family tree of a patient with autosomal-inherited AS, who had nephrotic syndrome as her first manifestation.

**Results:** The proband was a girl of 11 months who presented with nephritic and nephrotic syndromes including gross hematuria but had a normal renal function. Her treatment course was complicated by steroid resistance and a poor response to cyclosporine A and cyclophosphamide pulse therapy. Renal biopsy was performed 2 years after disease onset; light microscopy showed glomerular segmental mesangio-proliferative lesions, and type IV collagen staining showed the loss of the α3 chain in the glomerular and tubular basement membrane (GBM and TBM) and α5 chain loss in the GBM. Electron microscopy showed uneven GBM thickness, with the dense basement membrane (BM) layer obviously delaminated and torn, showing a typical “lace-like” change. The segmental BM was loosened and widened. Her father did not develop microscopic hematuria until 10 years later, while her grandmother had asymptomatic hematuria and proteinuria when the proband was diagnosed. We detected a new COL4A4 mutation in the proband, namely c.1715delG (p.G572Vfs ^*^ 81) in exon 24. Her father and grandmother carried the same mutation, but her mother and sister did not.

**Conclusions:** We found a new potentially pathogenic mutation of COL4A4 in a patient with autosomal-inherited AS, which presented as nephrotic syndrome in infancy.

## Introduction

Alport syndrome (AS) is one of the most common hereditary kidney diseases, mainly manifesting as hematuria, proteinuria, and progressive renal failure (1). AS can be accompanied by sensorineural deafness and ocular anomalies (1). The pathogenesis of AS involves the mutation of the glomerular basement membrane (GBM) type IV collagen α3, α4, and α5 chains (2, 3). Three modes of inheritance exist for AS: X-linked Alport syndrome (XLAS) (mutations involving COL4A5 on the X chromosome), autosomal recessive Alport syndrome (ARAS) (mutations in COL4A3 or COL4A4 on chromosome 2), and autosomal dominant Alport syndrome (ADAS) (mutations in COL4A3 or COL4A4) (1, 3). XLAS is the most common form, and is found in 80–85% of patients with AS (3, 4), followed by ARAS and finally ADAS in <5% (5–9). With the advancement in kidney pathology interpretation and technological improvements in electron microscopy and genetic diagnosis, the understanding of AS has increased and more cases of AS are recognized. It is now known that patients with ADAS have milder symptoms with a median age of 17 at proteinuria onset, and rarely have nephrotic syndrome (5). The age of onset for end-stage renal disease (ESRD) in patients with ARAS is 21.8–25 years on average (10, 11). In Chinese patients with ARAS, nephrotic-range proteinuria was detected in 41% of patients between the age of 20 months and 27 years (median 11 years) but none of them developed ESRD (12). We herein report the clinical data, mutation analysis findings, and family tree of a patient with autosomal-inherited AS and nephrotic syndrome in infancy as the initial manifestation, who developed ESRD at 11 years.

### Patients

The proband (IV-1) was a 17-month-old girl admitted to our hospital on December 31st, 2008 with the initial presentation of foamy urine for 6 months. Foamy urine and gross hematuria occurred initially when she was 11 months old and she did not receive an in-depth examination until 7 days prior to the current admission. Urinalysis at a local hospital revealed 3+ proteinuria and 3+ occult blood. There was no relevant medical history reported, and the random urinalyses results for her parents were negative. On admission, her physical examination showed a body temperature of 36.3°C, a pulse rate of 120/min, a respiration rate of 28/min, and a blood pressure at 85/50 mmHg. Minor pharyngeal congestion without tonsil swelling was noted. Cardiac, pulmonary, and abdominal examinations were normal without edema. Laboratory tests after admission showed 3+ proteinuria, 3+ occult blood, and a urinary red blood cell count of 5,493.2/μL. Her daily urine protein excretion amount was 150 mg/Kg and the spot urine protein/creatinine ratio was 6.9 mg/mg. Other serum biochemistry results were as follows: albumin 22.5 g/L, total cholesterol 4.58 mmol/L, urea nitrogen 2.09 mmol/L, and creatinine 41.0 μmol/L. Her complement 3 (C3), C4, immunoglobulin A (IgA), IgM, and IgG levels were normal. Computed tomography of the kidneys did not show any structural abnormalities. She received an initial diagnosis of idiopathic nephrotic syndrome, since she did not receive a renal biopsy. However, she failed to achieve remission after 4 weeks of prednisone (60 mg/m^2^) use and her response to pulsed methylprednisolone, cyclosporine A, and pulse cyclophosphamide was poor.

On November 8th, 2010, she received a renal biopsy. The results showed that 53 out of 54 glomeruli had slightly widened mesangial regions, with open capillaries, occasional paired endothelial cells, and segmentally layered capsule walls. Periodic Acid-Schiff Methenamine (PASM)-Masson staining was negative. In addition, her tubular epithelia were flat with focal border shedding. A small number of protein and erythrocyte casts were present in the tubular lumen, accompanied by focal tubular atrophy, basement membrane (BM) thickening, surrounding stroma fibrosis, and clustered foam cell aggregation. Multinucleated giant cells were occasionally observed with focal infiltration of mononuclear cells, along with hyaline degeneration of the arteries. Type IV collagen staining showed the absence of α3 on the glomerular BM (GBM) and tubular BM (TBM) as well as the absence of α5 on the GBM. Immunofluorescence staining showed diffuse positive IgM with granular deposition in the mesangial areas and vascular crests, but the results were negative for IgG, IgA, C3, and C1q. Electron microscopy showed that the mesangial areas were not significantly widened, nor did they contain any electron-dense deposits. The glomerular capillaries were open, but the BM thickness was uneven, ranging from 145–440 to 600–700 nm, with irregular inner and outer edges. The dense layer of the BM was prominently layered and torn, showing typical “basket weave” changes, and the segmental BM was loose and widened. The podocyte cytoplasm contained a few microvilli, with widely fused foot processes (80–90%). Part of the TBMs also showed stratification and tearing. These pathological findings were consistent with AS.

**Figure 1:**
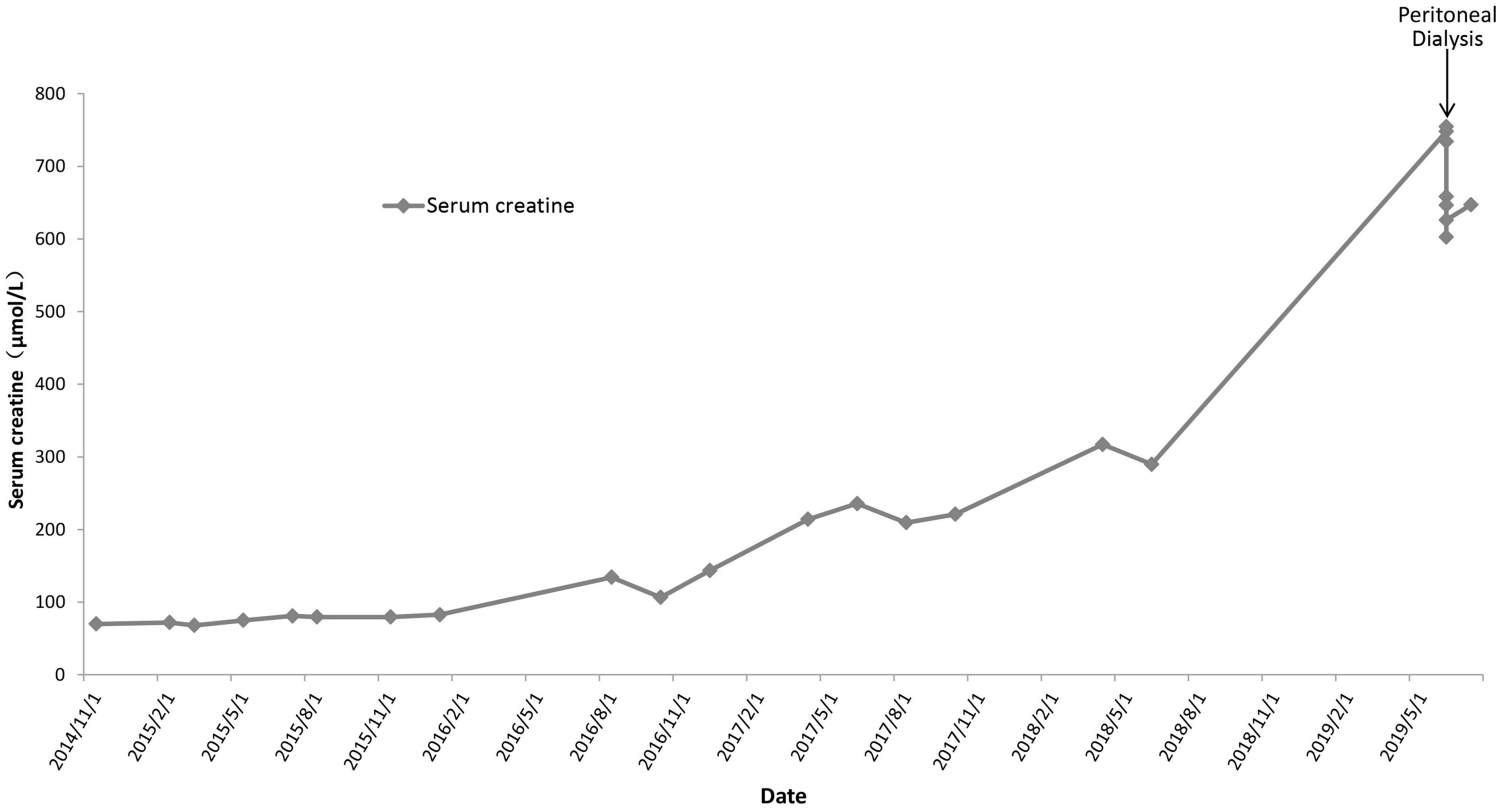
The temporal trends of serum creatinine for the proband.

Based on her clinical manifestations and pathological characteristics, a diagnosis of AS was made. She was then given angiotensin-converting enzyme inhibitors for treatment. Follow-up urinalysis showed persistent 3+ occult blood, 2+ to 3+ proteinuria, and red blood cell counts between 215.50 and 5787.70/μL. Her serum creatinine levels increased gradually (Figure 1). Eleven years after her initial presentation, she developed ESRD and started peritoneal dialysis on June 14th, 2019.

This study was approved by the Ethics Committee of The Affiliated Hospital of Qingdao University. All procedures performed in studies involving human participants were in accordance with the ethical standards of the institutional and/or national research committee and with the 1964 Helsinki declaration and its later amendments or comparable ethical standards. Written informed consent was obtained from the participant's legal guardian.

### Clinical Data of the Proband's Family

We clarified the family history of the proband among her relatives within two generations and constructed a family tree. The information collected included age, gender, blood pressure, the presence or absence of edema, the integrity of their visual and hearing functions as well as their urinalysis and renal function test results.

**Figure 2:**
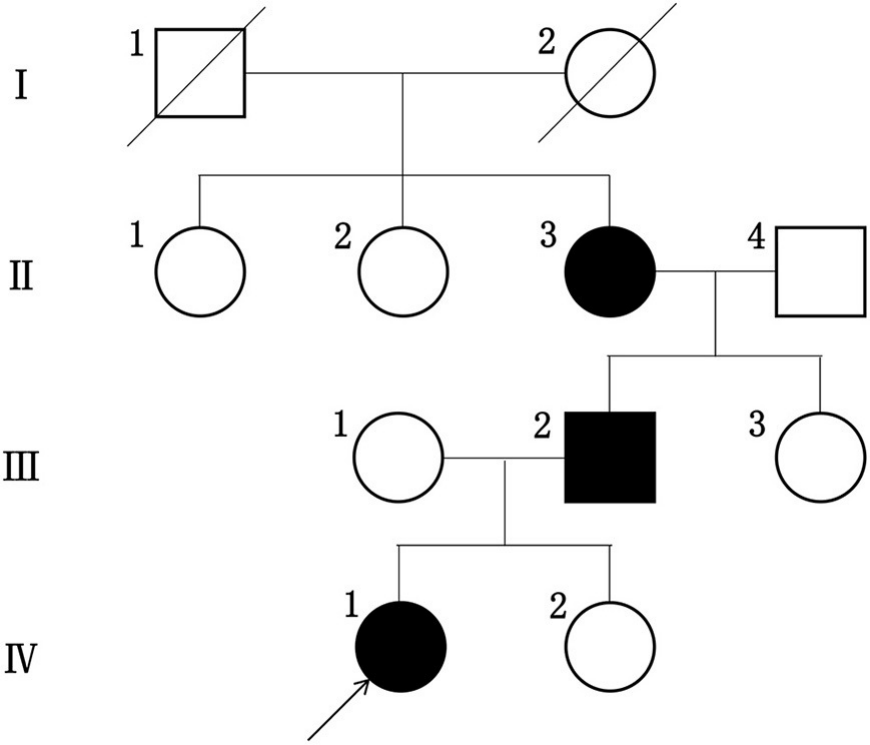
The pedigree tree of this proband.

The proband did not provide a detailed family history of kidney disease during her initial presentation and the urinalysis results for her parents obtained during the visit were normal. Therefore, we did not consider hereditary nephropathy as a potential explanation for her manifestations. Renal biopsy was later performed because her presumptive diagnosis of idiopathic nephrotic syndrome was refractory to available therapies. When the proband received the diagnosis of AS, her family members underwent repeat urinalyses. The results from her parents and little sister remained normal, but her grandmother was found to have asymptomatic hematuria and proteinuria at the age of 52, and her urinalysis showed 2–3+ proteinuria. Her father developed microscopic hematuria after 10 years of follow-up. None of her family members had hearing impairments or any visual defects. The proband's family tree is shown in Figure 2.

### Analysis of Gene Mutations

Peripheral blood samples were collected from each member of the proband's family with their consent and sent to MyGenostics Medical Laboratory (Beijing, China) for targeted next-generation sequencing (NGS) using a renal disease panel, followed by familial verification based on Sanger sequencing. We then proceeded to detect copy number variations (CNVs). Mutation Taster was used to predict the harmfulness of the mutations.

The proband was found to have a new deletional mutation involving COL4A4, specifically c.1715delG in exon 24, a heterozygous mutation. No evidence of CNVs was found. The same gene mutation was found in her father and grandmother, but no mutations were found in her mother and sister. This mutation was predicted to result in amino acid changes (p.G572Vfs ^*^ 81). A prediction based on the Mutation Taster suggested that the amino acid site change caused by this mutation was pathogenic.

## Discussion

AS is known as hereditary progressive nephritis (3) and is caused by damages to the BM because of mutations involving the type IV collagen α chain. Type IV collagen, a vital component of GBM, consists of α1–α6 chains, encoded by COL4A1 to COL4A6. With the advancement of mutation detection technology based on NGS, individuals with autosomal-related AS are increasingly diagnosed (4, 13). Proteinuria is recognized in childhood, sometimes in the nephrotic range, in male patients with XLAS and in ARAS patients, while milder symptoms are found in ADAS patients (14). We reported a patient with autosomal-related AS who was misdiagnosed with idiopath1ic nephrotic syndrome in infancy and finally developed ESRD at 11 years.

The timing of renal biopsy is very important for treatment guidance and prognosis estimation. Indications for renal biopsy in children with nephrotic syndrome include steroid resistance, presentation for more than 1 year, persistent gross hematuria, a high index of suspicion for different underlying pathologies, etc. (15). The initial manifestation for this patient was nephrotic syndrome with gross hematuria, and steroid resistance was found later. Both nephrotic syndrome with gross hematuria and steroid resistance can be indications for renal biopsy. We suggested renal biopsy at the time of initial diagnosis, but her parents refused because the child was too young and they were concerned about the risks associated with anesthesia. When the patient was close to 4 years old, her parents finally agreed to renal biopsy, which was performed at another hospital. We regret that we could not obtain the original pathological samples for detailed examinations, which is one of the limitations of this case.

The proband in this report satisfied the diagnostic criteria for AS given in the practical guidelines for the condition (13); that is, she had persistent glomerular hematuria and proteinuria, developed ESRD after follow-up, had GBMs without collagen type IV α3 and α5 chains, with lamellated GBMs under electron microscopy, and COL4A4 mutations. Based on the presence of hematuria, a relevant family history, and the same COL4A4 mutation, her father and grandmother might also have hematuria or/and proteinuria. Three generations of the proband's family had manifestations of hematuria and the same COL4A4 mutation, leading to a diagnosis of ADAS. However, the type IV collagen staining result was negative for α3 on both the GBM and Bowman's capsule (BC), but was negative for α5 on the GBM and positive on the BC. This is the typical finding for patients with ARAS (14). Additionally, the clinical course of the disease was severe in the proband while the other two family members with the same mutation showed milder symptoms. Thus, we speculated that the patient had a compound heterozygous mutation rather than a dominantly inherited one. Therefore, NGS with a renal disease panel (including exon and reported introns) was performed and CNVs were detected, but only one mutation involving COL4A4 was found without CNVs. In some studies, patients with one mutation on CLO4A3 or CLO4A4 were diagnosed with ADAS, even though they had severe disease at a younger age and typical features of AS. However, it remains unknown if these patients had a second mutation that was never detected (16). About 10% of patients with clinically and pathologically confirmed AS have mutations that are not identified by NGS or whole exome sequencing (17). We think this proband might have another variant of the maternal allele but could not detect it because it may have been an intronic variant.

The presentations of AS vary widely with different genetic patterns. The most common feature is hematuria. Proteinuria appears at an early stage of childhood in male XLAS patients, sometimes in the nephrotic range, and 90% of patients develop ESRD by the age of 40 years, with the median age of ESRD development being 25 years (18). Patients with ARAS show symptoms similar to those of male XLAS patients and patients with ADAS have milder manifestations than those with other AS types (5). A systematic review of 777 thin basement membrane nephropathy/ADAS patients with a single mutation (COL4A3/COL4A4) showed that the prevalence of ESRD in this population was 15.1%, compared to a prevalence of 62% in patients with ARAS and 70% in male patients with XLAS. The median age at ESRD onset was 55 years, while the ages of onset in patients with ARAS and male patients with XLAS were 21 and 25 years, respectively (16). The proband in this report developed nephrotic syndrome with gross hematuria in infancy, and she had a poor renal prognosis. Initially, her renal function was normal but deteriorated over time. Her parents had normal urinalysis results initially, but her father developed microscopic hematuria nearly 10 years later. Her grandmother was found to have asymptomatic hematuria and proteinuria. We speculate that her father and grandmother were carriers of ARAS with milder symptoms. Currently, no clear genotype-phenotype association has been observed (2). Furthermore, this clinical heterogeneity cannot be explained solely by genetic mutations, but may be related to differences in penetrance, environmental factors, or other unknown genetic differences (5). Epigenetic factors including DNA methylation, histone modification, chromatin remodeling, and non-coding RNA regulation may also be contributory. Researchers have proposed a double gene theory to explain heterogeneity in other diseases (19), but none addresses AS.

In our report, all involved cases had a frameshift mutation in exon 24 of COL4A4, with a guanine deletion at position 1,715 in the coding region (c.1715delG), resulting in the change of glycine to valine and the premature appearance of the stop codon. This change resulted in protein truncation (p.G572Vfs ^*^ 81). Mutation Taster also predicted that the amino acid site change was harmful. We believe that the mutation is pathogenic in this pedigree, and after consulting ClinVar, Single Nucleotide Polymorphism database (dbSNP), Varcards, Human Gene Mutation database (HGMD), Online Mendelian Inheritance in Man (OMIM), and Pubmed, we believe this new mutation may be the first reported case.

The mode of inheritance for the same pathogenic mutation may differ. Mutations previously thought to be autosomal recessively inherited can also be inherited autosomal dominantly. Five types of mutations in 16 ADAS families were deemed pathogenic in previous reports on ARAS, and, interestingly, the parents of heterozygous carriers of these mutations are usually asymptomatic or only exhibit microscopic hematuria (5). In our study, only the proband had clinical symptoms initially. Although the mutation patterns were heterozygous, the mode of inheritance could not be clarified despite a positive family history based on her father and grandmother. This phenomenon is a reminder that patients may present early when their family members are asymptomatic or have only mild manifestations. Therefore, it is necessary to adequately consider whether the inheritance is dominant or recessive.

## Conclusions

The clinical manifestations and course of disease development for the patient in this report suggest that for children with atypical symptoms or refractoriness to conventional treatment, kidney biopsy should be performed promptly to determine the underlying pathology in order to facilitate subsequent management. Genetic testing may also be necessary. It is prudent to complete a pedigree investigation and arrange long-term follow up for patients with hereditary nephropathy, since the phenotypes of different patients with the same mutation can vary greatly.

## Data Availability Statement

The raw data supporting the conclusions of this article will be made available by the authors, without undue reservation.

## Ethics Statement

The studies involving human participants were reviewed and approved by Ethics Committee of The Affiliated Hospital of Qingdao University. Written informed consent to participate in this study was provided by the participants' legal guardian/next of kin.

## Author Contributions

DW: conceptualization, data curation, formal analysis, software, and writing—original draft. CS: methodology, project administration, resources, software, and visualization. XJ: data curation and investigation. QZ: conceptualization, formal analysis, project administration, resources, supervision, and editing. HC: data curation and methodology. YL: conceptualization, formal analysis, project administration, supervision, validation, and writing—review. All authors contributed to the article and approved the submitted version.

## Conflict of Interest

The authors declare that the research was conducted in the absence of any commercial or financial relationships that could be construed as a potential conflict of interest.

## Publisher's Note

All claims expressed in this article are solely those of the authors and do not necessarily represent those of their affiliated organizations, or those of the publisher, the editors and the reviewers. Any product that may be evaluated in this article, or claim that may be made by its manufacturer, is not guaranteed or endorsed by the publisher.

## Abbreviations

AS: Alport syndrome
GBM: glomerular basement membrane
TBM: tubular basement membrane
BM: basement membrane
XLAS: X-linked Alport syndrome
ARAS: autosomal recessive Alport syndrome
ADAS: autosomal dominant Alport syndrome
ESRD: end-stage renal disease
C3: complement 3
Ig: immunoglobulin
PASM: periodic Acid-Schiff Methenamine
NGS: next-generation sequencing
CNVs: copy number variations
BC: Bowman's capsule
dbSNP: Single Nucleotide Polymorphism database
HGMD: Human Gene Mutation database
OMIM: Online Mendelian Inheritance in Man.
